# Supplementary material for: Study on the Correlations between Quality Indicators of Dry-Aged Beef and Microbial Succession during Fermentation
Source: Foods. 2024 May 16;13(10):1552. doi: 10.3390/foods13101552 (PMC11120345; doi:10.3390/foods13101552)
Supplement: Supplementary file 1 [file foods-13-01552-s001.zip › foods-2995236-supplementary.pdf]

Journal: *Foods*

Manuscript Title: Study on the correlations between quality indicators of dry-aged beef and microbial succession during fermentation

Authors: Yuliang Cheng, Yiyun Meng, Lin Xu, Hang Yu, Yahui Guo, Yunfei Xie, Weirong Yao, He Qian

Affiliations and Addresses: State Key Laboratory of Food Science and Resources, School of Food Science and Technology, Collaborative Innovation Center of Food Safety and Quality Control in Jiangsu Province, Jiangnan University, Wuxi 214122, PRChina

Correspondence to: ylcheng@jiangnan.edu.cn (CYL).

Table S1 Analysis of key flavor compounds of the dry-aged beef

Flavor compounds	Species	Odor characteristics [29-30]	Sensory threshold (μg/kg)	ROAV				
				0 d	3.5 d	7 d	14 d	21 d
aldehydes	Hexanal	Fragrance of grass, fruit, leaf	4.5	—	0.11	0.45	0.99	0.44
	Heptanal	Fruit, milk, soap	3	0.94	3.27	5.02	—	2.17
	Octanal	Citrus, lemon, honey, Aroma of fruit, wax, fresh grass	0.8	10.10	17.97	11.70	10.86	14.33
	Nonanal	Fragrance of flowers, citrus, grass, wax, fat	1	100.00	100.00	40.99	53.38	100.00
	Furfural	Aromas of wood, caramel, baked goods	3	0.20	0.27	—	—	—
alcohols	Pentanol	Mild fruit and spice aromas	0.15	—	14.41	100.00	100.00	27.33
	1-Octanol	Strong floral, citrus, walnut, moss notes	110	0.08	0.10	0.13	0.11	0.09
	Oct-1-en-3-ol	Mushroom, earth, grass, lavender, rose	1	2.16	3.75	9.73	6.55	6.24
alkenes	Styrene	Sweet scent, floral scent, sesame oil scent	47	0.14	0.01	—	0.54	0.05
ketones	Acetoin	Strong notes of milk, butter, mushroom, and vanilla	55	0.85	0.30	3.25	6.45	0.67
esters	Vinyl Hexanoate	Ester flavor	7.5	0.29	0.33	1.80	—	—
furans	2-Amylfuran	Fresh, meaty, mung bean, butter	6	0.31	0.70	1.09	0.80	0.24

Table S2 Effective sequence statistics of bacteria and fungi

Sample	Bacteria		Fungi	
	The number of effective sequences	The length of effective sequences (M)	The number of effective sequences	The length of effective sequences (M)
0 d	65490	27.28	80432	22.02
3.5 d	67267	28.38	80452	21.77
7 d	62268	26.53	79890	21.77
14 d	56089	23.94	75915	21.08
21 d	67244	28.70	80477	21.72

Table S3 Diversity analysis of bacterial community during the dry aging process

Sample	Shannon	Simpson	Chao1	Goods _coverage
0 d	6.42	0.97	279.00	1.00
3.5 d	4.58	0.87	162.05	1.00
7 d	2.78	0.65	39.83	1.00
14 d	3.64	0.87	34.33	1.00
21 d	2.64	0.65	37.33	1.00

Table S4 Diversity analysis of fungi during the dry aging process

Sample	Shannon	Simpson	Chao1	Goods_coverage
0 d	3.52	0.79	77.00	1.00
3.5 d	2.76	0.69	56.00	1.00
7 d	1.87	0.49	50.67	1.00
14 d	2.72	0.71	41.67	1.00
21 d	1.59	0.44	39.00	1.00
